# Teachers’ emotions in the time of COVID: Thematic analysis of interview data reveals drivers of professional agency

**DOI:** 10.3389/fpsyg.2022.987690

**Published:** 2022-11-03

**Authors:** Karen Porter, Paula Jean Miles, David Ian Donaldson

**Affiliations:** School of Psychology and Neuroscience, University of St. Andrews, St. Andrews, United Kingdom

**Keywords:** teaching, individual differences, education, agency, emotion, emotion percept, complex dynamic systems, thematic analysis

## Abstract

**Purpose:**

We explored two complex phenomena associated with effective education. First, teachers’ professional agency, the volitional actions they take in response to perceived opportunities, was examined to consider individual differences in its enactment. Second, “strong” emotions have been proposed as important in teaching and learning, and we wished to clarify which basic emotions might be involved, besides curiosity, which is a known emotional factor in engagement in teaching. We also explored how agency and basic emotions might be related.

**Approach:**

Thirteen teachers working in Scottish secondary schools were interviewed at the start of the covid pandemic in 2020 to discuss relevant feelings, thoughts and actions arising from unprecedented changes in their lives and professional practices. Thematic analysis was used to identify aspects of agentic behavior and basic emotions expressed.

**Findings:**

Teacher agency was expressed through adaptability, collective agency, constrained agency, and non-action. Four basic emotion percepts were identified, which we label as “CARE”, “CURIOSITY”, “COOPERATION”, and “CHALLENGE”.

**Originality:**

We extend the definition of agency to include volitional non-action as a response to opportunity. In contrast to prior research emphasizing emotions as an outcome of volitional behavior, we explore emotions preceding agency. We develop four theoretical propositions related to teacher emotions. (1) Four emotion percepts substantially influence teachers’ voluntary motivated behavior. (2) The amount and proportion of emotions experienced varies between individual teachers. (3) The four percepts are experienced concurrently or in rapid succession in engaged teaching contexts. (4) Professional experience and specific situational factors also influence teachers’ behavioral choices. For future consideration, we suggest that awareness of emotion percepts may encourage both teachers’ engagement and their professional agency for the benefit of their pedagogical practice and outcomes for their students.

## Introduction

The commitment that humans make to teaching is unique. Despite the prevalence of social *learning* behavior, few other species have been found to demonstrate *teaching*, changing their behavior at a cost to themselves to support a learner in acquiring new skills or knowledge (Caro and Hauser, 1992; Kline, 2015). Many of our own species, on the other hand, intentionally devote significant amounts of their limited resources to transmit cumulative cultural capital to others. Research investigating teaching has largely focused on cognitive phenomena and behavioral outcomes, notably neglecting close examination of the role of emotions in teachers’ volitional engagement in their chosen profession (Carson and Chase, 2009; Han and Yin, 2016), despite growing recognition of the role played by emotions in teaching and learning (Pekrun et al., 2007; Keller and Becker, 2021). The research reported in the current paper therefore seeks to expand on the question “*what motivates us to teach?*” with an assumption that there is a significant role for basic emotions in addition to cognitive and behavioral factors, recognizing that relationships between factors involved in teaching constitute complex dynamic systems. Before detailing the research methods and findings, we offer context by expanding on theories about teacher agency, and on current accounts of the role emotion plays in teaching.

### Existing theories of teacher agency

Teacher agency remains poorly conceptualized and under-theorized, despite growing recognition of its value in education settings (Emirbayer and Mische, 1998; Priestley et al., 2015; Aspbury-Miyanishi, 2022). The lack of consensus is partly due to recognition that teacher agency is a complex multilevel construct, often considered in conjunction with other constructs such as identity and power (Impedovo, 2021; Sherman and Teemant, 2021). Nevertheless, we take as a starting position Sherman and Teemant’s summary of agency as “action taken not accidentally, unknowingly, unwittingly or unwillingly” (2021, p. 5). A recurring consideration of teacher’s agency has been the influence of the social context in which it is experienced, including factors such as available resources, relations with others (Impedovo, 2021), policies, norms, language, and support structures (Lasky, 2005), as well as relationships between teachers, their colleagues, pupils, and parents (Pyhältö et al., 2015).

Reasons proposed for the recent growing interest in teachers’ agency may appear contradictory (Erss, 2018). On the one hand is the perception that recent education initiatives necessitate teachers’ active engagement in collective issues requiring change, such as shaping pedagogical practices, curriculum reform, and social progress (Pyhältö et al., 2014; Biesta et al., 2015; Priestley et al., 2015). Individually, teachers are also required to be “horizon workers” (Tateo, 2021, p. 93), responding dynamically to their students’ ongoing progression and their own professional development. Indeed, teachers subject to fewer constraints imposed by rigidly defined curricula (Sang, 2020) are assumed to be acting in response to their own values and goals rather than simply delivering centrally mandated curriculum content (Scottish Executive, 2006b). On the other hand, education reforms have also made teachers accountable for delivery of practices, such as standard tests, to which they may have had no input, resulting in perceptions of being deprived of professional standing, collective power, and individual agency (Sahlberg, 2010; Tao and Gao, 2017; Benesch, 2018). Combining these two viewpoints, personal history, school contexts and the prevailing political milieu unite in complex ways to affect teachers’ experience of agency, both from a bottom-up perspective driven by the individual’s values, and from a top-down socio-cultural perspective involving a web of intricate interactions.

Compared to the sociocultural and temporal focus on teacher agency, relatively little effort has been focused on individual differences in teachers’ experience of agency. Although MacLean (2016) concurs with the notion of teacher agency as an emergent phenomenon, she proposes that it develops through a process of reflective, individualized practice. By contrast, Aspbury-Miyanishi (2022) argues that, although teachers may retrospectively ascribe actions to be the result of conscious choices, in fact experienced teachers recognize themselves as making few conscious choices. Instead, they act in the only way they consider appropriate to the circumstances in which they find themselves. From a theoretical perspective therefore, a clear gap exists in our understanding of how teachers differ in their enactment of agency.

### The role of emotion in education

Emotions are increasingly recognized as integral to engagement in teaching and learning, and to teachers’ experience of agency when contentious situations arise (Benesch, 2018). Research into the role of emotions in workplaces has been limited compared to more general contexts (Hökkä et al., 2019), with education settings forming a subset of such research, and a still smaller body of work specifically focused on teachers’ rather than students’ emotions (e.g., Hannula, 2012; Frenzel, 2014; Xu, 2018; Henritius et al., 2019; Sun and Yang, 2021). Relations between teachers’ and students’ emotions have been investigated in both directions. Effects of teachers’ emotions on students include the impact on Students’ emotions and perceptions, competence, motivation, use of regulatory strategies, academic performance, discipline, and social behavior (Fried, 2010; Rodrigo-Ruiz, 2016). Conversely, teacher emotions are impacted by students’ academic performance, behavior, student-teacher relationships, students’ reported enjoyment and the perceived effectiveness of support they receive (Frenzel et al., 2007; Scott and Sutton, 2009; Beilock et al., 2010; Klassen et al., 2012; Chang, 2013). Students believe that good teachers nurture their wellbeing, despite researchers’ ongoing issues with how to measure “goodness” (Usher, 2021).

In practice, teachers’ emotions are proposed as resulting from appraisal and evaluation of multi-component relationships (Reschly and Christenson, 2012; Frenzel, 2014; Reeve and Su, 2014). Although there is recognition in existing literature of reciprocity between cognitive constructs (such as goals, perceptions, and appraisals), and teachers’ emotions (Baird et al., 1990; Frenzel, 2014), examination of relationships between these constructs has typically considered the impact of cognitions on emotion (Chang, 2013). Specifically, Control-Value Theory proposes that emotions result from appraisal of the perception of control one has, and the value assigned to specific goals and situations (Pekrun and Perry, 2014). In the specific case of teachers, Frenzel (2014) proposes a model that links emotions with antecedents such as perception of student behaviors, goals for student behaviors, and appraisals of student and teacher performance and progress toward meeting goals. The resulting achievement emotions include enjoyment, pride, enthusiasm, anger, anxiety and shame, depending on appraisal of outcomes (Frenzel, 2014; Owens and Hudson, 2021). Similarly, beliefs about self-efficacy have been related to resulting emotions and the degree of effort teachers subsequently expend (Jarrell et al., 2017; Harley et al., 2020). The focus to date on teachers’ emotional outcomes contrasts with the dearth of research on routes by which teachers’ emotions impact their beliefs and actions.

### Distinguishing basic and secondary emotions

In broad terms, emotions are complex constructs incorporating neural, physiological and behavioral responses to stimuli, together with subjective responses to the experience (Celeghin et al., 2017). A contentious debate continues about whether humans experience basic emotions that are distinguishable from secondary emotions (Ortony, 2021; compare Fossati, 2012; Feldman-Barrett, 2017). Secondary emotions are distinguished by their abundance and by presumed complex processes involving cognitive appraisal of situations that gives rise to their experience (Braniecka et al., 2014; Linnenbrink-Garcia and Barger, 2014). One set of criteria for basic emotions includes the presence of distinct biological mechanisms closely associated with specific behavior, thought and memory responses, characterized by distinct physiology and physiognomy, early ontogeny and phylogeny, and rapid arousal and short duration (Ekman, 1992). Theoretical accounts disagree, among other things, on the number of basic emotions proposed, which some argue do not correlate directly with what they term “survival circuits” (Panksepp et al., 2017, p. 192). In addition to biological definitions of what constitutes a basic emotion, Celeghin et al. (2017) also offer categorical and psychological definitions of basicness. Categorical definitions incorporate sufficient criteria to allow specific examples to be confidently included in, or excluded from that category, for example “trees” incorporates oak and ash, while excluding daffodil. For present purposes, therefore, basic emotions are defined as referring to only a single emotion, as distinct from combinations of single emotions or combinations of emotion and cognition (Celeghin et al., 2017).

In education settings, teachers’ curiosity has been shown to impact relationships with students to a small but significant extent (Amorim Neto et al., 2020). For example, Izard (2007) identified curiosity as a ubiquitous quality that sustains active engagement in exploration, improved learning, information retrieval and behavior choices (see also Grossnickle, 2016; Oudeyer et al., 2016). Experiencing curiosity also allows humans to benefit from their own and others’ experiences, passed on through cultural knowledge (Silvia, 2017). Curiosity is also considered to be a fundamental emotion driving and maintaining cognitive system development (Buck, 2014). Consistent with the comments about emotion above, there is also no universally agreed definition of curiosity (Pekrun, 2019), nor is it always clear when potential synonyms such as openness, interest and seeking are being used to describe the same phenomenon (Grossnickle, 2016). Nonetheless, curiosity is considered as an adaptive emotion that motivates learning (Alcaro and Panksepp, 2011), distinct from cognitive or behavioral phenomena (Pekrun and Linnenbrink-Garcia, 2014; Ryan and Deci, 2017). Defining curiosity as a basic emotion therefore acknowledges biological processes distinct from cognitively derived states that prime autonomous seeking behavior (Ainley and Hidi, 2014). For example, a study of the association between curiosity and agency found energetic engagement with novel or ambiguous situations was associated with a willingness to wait and, rather than being given the right answer, a preference for hints that allow a correct solution to be found through one’s own effort (Metcalfe et al., 2021). Findings of a direct association between curiosity and volitional effort highlights the importance of considering how the experience of curiosity and other basic emotions is associated with teachers’ agency, complementary to research focused on cognitive appraisals giving rise to complex emotions that in turn influence autonomous behaviors (Schutz et al., 2007; Braniecka et al., 2014; Frenzel, 2014; Pekrun and Perry, 2014; Feldman-Barrett, 2017).

### Motivation for the current study

**FIGURE 1:**
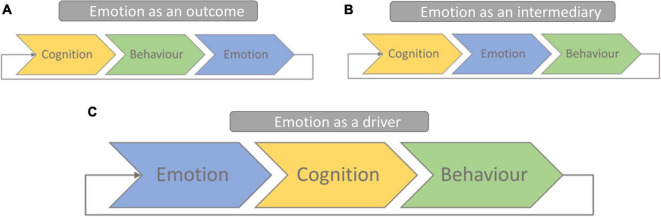
Existing theories of learning recognize reciprocal interactions between multiple factors, typically emphasizing routes to action that begin with cognitive constructs **(A,B)**. Here we propose an alternative framework emphasizing routes to action that begin with emotion **(C)**.

As the preceding review makes clear, we recognize reciprocal causality between various constructs that interact in complex dynamic systems to give rise to teachers’ agency. Crucially, the current study diverges from the existing literature in one key way, namely in how the complex processes that we examine are related. As illustrated in Figures 1A,B, existing theories typically begin with cognitive constructs when describing routes to the experience and enactment of agency, viewing emotion as either an experience that results from behavior (Figure 1A) or as a way of influencing behavioral outcomes (Figure 1B). Whilst we would not disagree that both viewpoints provide a useful way of understanding emotion, here we offer a distinctly different perspective. Specifically, the research reported in the current paper aims to examine the role that emotions play in the generation and enactment of agentic behavior (Figure 1C), viewing emotion as a driver of cognition and behavior, rather than simply an outcome (Damasio, 2006).

In addition to exploring how curiosity might relate to the initiation of agency in teachers, we considered that other emotions may also influence teachers’ expression of agency, given earlier reports of a role for unspecified “strong” emotions in education (e.g., Danvers, 2015; Tillmanns, 2019). Indeed, we consider the lack of both empirical research and theory development about the role of emotions in teaching to be surprising given the prevalence of emotions in the classroom (Pekrun and Linnenbrink-Garcia, 2014). Consequently, we address the question of how teachers report the experience of emotions in the classroom by exploring the following hypotheses:

• Teachers report experiencing agency in the course of their professional lives.
• Teachers experience a range of emotions that influence their expression of agency during teaching, incorporating a small number of distinct emotions including, but not limited to, curiosity.
• Individual differences in the profile of teachers’ emotional experiences are reflected in the number and proportion of emotions observed.

## Materials and methods

### Research approach

We employed a broadly constructivist research paradigm using grounded theory. Grounded theory was selected in view of its suitability for exploring hypotheses and constructing theories, in contrast with postpositivist and positivist paradigms (Oktay, 2012). Our starting position was the hypothesis that a teacher’s curiosity relates to their experience of agency (Metcalfe et al., 2021), and our theoretical aim was to explore whether additional basic emotions or biological drives would also be involved in developing and experiencing agency.

Interviews were conducted by the lead author, a Ph.D student whose interest in psychological factors associated with teachers’ experience of, and expression of, professional agency arose from previous roles managing change projects for commercial and educational organizations, during which she had experienced the significance of emotions in participants’ responses to disruption. The lead author’s aim was to address both idiographic and nomothetic perspectives on data collected to explicate individuals’ experiences and to establish what range of emotions might operate within a population of teachers. Although experienced in a variety of data analysis techniques, the lead author was unacquainted with Nvivo software (V12, QSR International, 2020), and therefore required a period of training prior to analyzing the transcribed data.

### Context

Education reform implemented in Scotland from 2010 onward has sought to achieve improvements in educational attainment through teachers’ reflective practices. The Curriculum for Excellence (CfE) aims to empower teachers to think and share ideas about their goals and values, and about their professional practice (Scottish Executive, 2006a), although movement from an “intended to an implemented curriculum” (Sinnema et al., 2020, p. 182) has been criticized as being slow. Scotland aligns with education reform internationally in shifting from the “top-down” control of curricula to prioritizing teachers’ agency, particularly their capability to design and deliver flexible curricula aligned with the needs of their school and classroom. The Curriculum for Excellence prioritizes outcomes in the form of children and young people becoming successful learners, confident individuals, responsible citizens and effective contributors rather than focusing on specific curriculum content (Hedge and MacKenzie, 2016). Critics have, however, identified incongruity between declarations of support for teacher agency, and subsequent narrow definitions of expected experiences and outcomes that promote compliance at odds with teachers’ autonomy (Priestley et al., 2015).

Following a decade of ongoing curriculum change then, participants in the current study faced specific challenges as schools abruptly closed, with teaching and learning redirected to online channels with immediate effect (Swinney, 2020). Sudden fundamental changes in teaching practices, and concern over their own and others’ wellbeing, meant unparalleled challenges to teachers’ roles as educators. Increased opportunities to exercise agency therefore arose from the need to develop innovative practices to augment or replace previously dominant approaches (Tsui et al., 2020), while the resultant upheaval was assumed to elicit a range of emotions and beliefs in relation to their own and their students’ unprecedented experiences (Narayanan and Ordynans, 2022). Teachers were uniquely placed to articulate their experiences of agency, motivation and internal emotional landscape since they are assumed to be reflective practitioners in their professional role as change agents for learners (GTCS, 2020).

Thirteen teachers were recruited using opportunistic sampling through an advertisement on professional support groups on Facebook whose members were primarily teachers in Scottish secondary schools (serving 12–18-year-olds). Secondary teachers were targeted since, as well as sharing challenges with teachers of other stages, they faced additional issues in respect of students scheduled to take crucial external exams in the near future. Potential participants contacting the lead author were provided with a participant information sheet giving details of what was required, inclusion criteria, and a consent form. All respondents subsequently agreed to participate in an interview, and none were excluded through failing to match inclusion criteria. Consent forms were either returned prior to the interview, or acknowledged and verbally agreed to at the start of the interview. All interviews were conducted between April and June 2020. The authors analyzed developing themes as each interview concluded and determined that data saturation was reached when analyses no longer introduced new themes, although rich data was still being found (Guest et al., 2016). Ethical approval for the study was granted by the School of Psychology and Neuroscience Ethics Committee, University of St. Andrews (approval code PS14861).

### Data collection methods

Having confirmed consent and collected brief biographical details, the interviewer explained that the scope of the discussion included participants’ emotions, thoughts and behaviors in response to recent events, and invited the participant to begin with whichever of the three they were most comfortable. Where necessary the interviewer referenced the topic guide (Supplementary Appendix 1) to ensure all three were discussed, following a semi-structured hierarchical focused format (Bernard, 2013). An example of a prompt related to actions is “In what ways have your actions changed recently, and how have they remained the same or similar to previously?” Related to thoughts, we asked “What consideration have you given to what is possible and what is no longer possible in the changing circumstances?” A prompt related to feelings is “What is your recent experience of levels of energy and ability to focus?” All interviews were conducted using Microsoft Teams (Version 1.5.00.21668) and were recorded for analysis with participants’ consent. Interviews lasted between 35 min and 1 h 55 min, with a median length of 1 h. In all cases the interviewer and interviewees were alone in a room in their homes since they were subject to lockdown restrictions. On a few occasions there were brief interruptions from family members, specifically children of interviewees asking questions about domestic situations. Textual analysis was facilitated by the use of Nvivo software (V12, QSR International, 2020).

### Units of study

Participants included a teacher of students with special education needs, as well as teachers of English, mathematics and science, history and art. Teaching experience ranged from 4 years to over 30 years, with four participants having entered teaching following careers in unrelated fields. Eight participants were female and five were male, consistent with the typical gender ratio in Scottish secondary schools (Scottish Government, 2019).

### Data processing and analysis

Each interview recording was transcribed into a separate file and all identifying information was anonymized/pseudonymized as appropriate. Recordings and all subsequent files used for analysis were stored securely in line with data security and retention guidance issued by the University of St. Andrews.

Since the scope of the current study extended to at least two complex phenomena, we adopted a transdisciplinary integrative approach to defining aims, selecting the unit of analysis, and selecting research methods (Hiver et al., 2022). Our aims were twofold: to investigate whether agency and curiosity were reported by our participants, as has been previously reported, and to explore whether other emotions were reported as relevant to participants’ professional lives.

The primary unit of analysis was individual interviews, with additional analysis conducted to confirm thematic findings by comparing individuals’ responses. Individual transcription files were analyzed using six phase thematic analysis (Braun and Clarke, 2006, 2013) to identify common and repeated emergent themes. Initial codes and themes were generated using a combined deductive/inductive approach aligned with phases 1–3 of Braun and Clarke’s approach. The deductive component arose from *a priori* assumptions that (a) teachers would report feelings of curiosity related to opportunities arising from recent novel circumstances, and (b) teachers would report behaviors that demonstrate their experience of professional agency. Inductive analysis was driven by interest in what other psychological phenomena, particularly emotions, would emerge from the data (Fereday and Muir-Cochrane, 2006). For phase 4, the first author and two independent coders met to discuss initial findings from the first six interviews, and reached agreement on theme descriptions, numbers and codes. The first author and one coder each then separately coded a transcript. During analysis, coders recognized that the same utterance could refer to more than one emotion and agreed that multiple codes should be assigned where relevant. Assigned codes matched in 87% of instances. A similar exercise with a second transcript and the second coder resulted in 85% agreement on codes assigned, indicating a high level of consistency in interpreting participants’ meaning. The first author completed coding on the remainder of the transcripts. Subsequent meetings between coders, and between all authors further clarified emergent themes, definitions and names of themes (phase 5). The final phase is addressed in the findings reported below (Braun and Clarke, 2013). In addition to textual analysis, we reference examples where gestures added to the communication of emotion states (Hostetter, 2011). Although we recognize the limitations of a tool initially aimed at biomedicine and health services (Buus and Perron, 2020), and the risk of formulaic interpretation of qualitative data (LaMarre and Chamberlain, 2022), nevertheless the Consolidated criteria for reporting qualitative research (COREQ) served as a valuable guideline for including relevant methodological detail in reporting findings (Tong et al., 2007, Supplementary Appendix 2).

## Results

Thematic analysis resulted in the emergence of key themes related to both agency and emotions. We identified four sub-themes related to the experience of agency and four themes related to distinct emotions. An *a priori* assumption that teachers would report behaving with agency was supported. Examples of participants’ expressions of agency are shown in Table 1, highlighting the dominance of adaptability as a sub-theme. The first three examples of agency in Table 1 exemplify adaptability at strategic, tactical and operational levels, respectively. Heather reported previously making a strategic decision when she seized an opportunity to leave a career in financial services and retrain as a teacher; Amanda made tactical efforts to change her behavior so that she continued to perform as an effective teacher; and Sheila described a specific circumstance in which she chose an unprecedented course of action (Greer, 1991; Goi, 2018).

**TABLE 1:** Examples illustrating the expression of agency for each teacher.

Examples of expressions of agency		
Participant	Quote	Sub-theme
Heather (F)	I decided this was my chance to go and do something different, so I took voluntary redundancy, did my teacher training and, here we are, a whole new career.	Adaptability
Amanda (F)	It’s a massive amount of change… and my attitude is “we will make it work” and it’s what I’m getting paid for and I will do my best.	Adaptability and collective agency
Sheila (F)*	I was so impressed with (a student) that I just … got him the equipment. I couldn’t do that for every single kid I teach.	Adaptability
George (M)	(In response to a job offer) I ended up not taking it because it just didn’t have the right feel for me… I thought, actually no, this is not for me. I’m actively looking (but) it’s not something I’m going to jump at.	Agentic non-action
Tony (M)	This was actually an opportunity for those of us that were champions of technology… (what I am) trying to do strategically in the school is … to empower teachers.	Adaptability
Gregory (M)	This is going to be a great opportunity for me to spend some time in professional development… because the one thing teachers never really get the chance to do is to spend a wee bit of time developing their craft.	Adaptability
Douglas (M)	I very much had to modify my goals…find other ways of making it light-hearted, so I’m not saying that it’s a challenge I’ve been meeting really but I have been trying.	Adaptability
Sandra (F)	We kind of decide what we want to do and … we work it so that we get, you know, basically what we want to happen. We kind of negotiate that, I suppose, by the way we work together.	Collective agency
Nanette (F)	I threw myself into trying to plan these lessons that would make sure the students were getting what they needed, outcomes-wise, but (also) had a bit of fun and really racking my brain on how to make that happen.	Adaptability
Emily (F)	I’ve been told by my boss this is what they need to do, but I know it’s too hard for them and I would never even do it in the classroom. So why am I expected to do it online with no support?	Constrained agency
Bethan (F)	I have had to change, as we all have had to… I pick up a bundle of newspapers every day. so that they have a fresh … national and regional paper. Every class begins with 10 min of reading the paper.	Adaptability
Carol (F)	(Recent circumstances have) shown me that whatever capacity I’m working in, I can do the job and I’m quite adaptable… We’re used to thinking outside of the box.	Adaptability
Jake (M)	I’ve had time to do things that I’ve never, I never would have had time to do before, just kind of experiment with different ways of teaching.	Adaptability

Sub-themes refer to codes assigned by the authors, including where multiple sub-themes were identified in the same text. *Technical difficulties with sound meant that only a minority of the interview with Sheila was transcribable.

Besides adaptability, themes of collective agency, constrained agency, and non-action are also illustrated in Table 1. Collective agency was illustrated by participants’ choices to engage in collaborative efforts such as subdividing large tasks among colleagues and then sharing results for the greater good of their students, colleagues, and themselves (Bandura, 2016). The agency shown by Emily was distinct from other participants in that, having perceived that there were no “good” choices open to her, she expressed a desire to have had other options available (Damman and Henkens, 2017). The label of “constrained agency” in these circumstances recognizes that whereas Sherman and Teemant (2021) include willingness to act in their definition of agency, Emily explicitly described reluctance to act or unenthusiastic inaction. In contrast, George’s decision not to take a job offer was a deliberate response after considering the opportunity offered, his future goals, potential alternatives, and his emotional “gut” response. His decision informs our proposed extension to Sherman and Teemant’s definition of agency in that his inaction was not accidental, unknowing, unwitting, nor unwilling (2021). We therefore propose an addendum to include intentional *inaction* as a possible expression of agency.

Thematic analysis also confirmed the *a priori* assumption that *curiosity* would be identified as a motivating emotion that underpinned agentic behavior. Crucially, *a posteriori* themes of *care*, *cooperation* and *challenge* also emerged. For the purposes of disambiguation, subsequent references will be to CURIOSITY, CARE, COOPERATION, and CHALLENGE, following the convention of using block capitals to distinguish themes related to emotions as descriptions of perceptual experience, and not simply lexical entities (Montag and Panksepp, 2017). We prefer the term *percept* over *concept* since percepts incorporate multiple modalities including thinking about and experiencing bodily feelings, and exhibiting actions associated with or exemplifying emotions.

**FIGURE 2:**
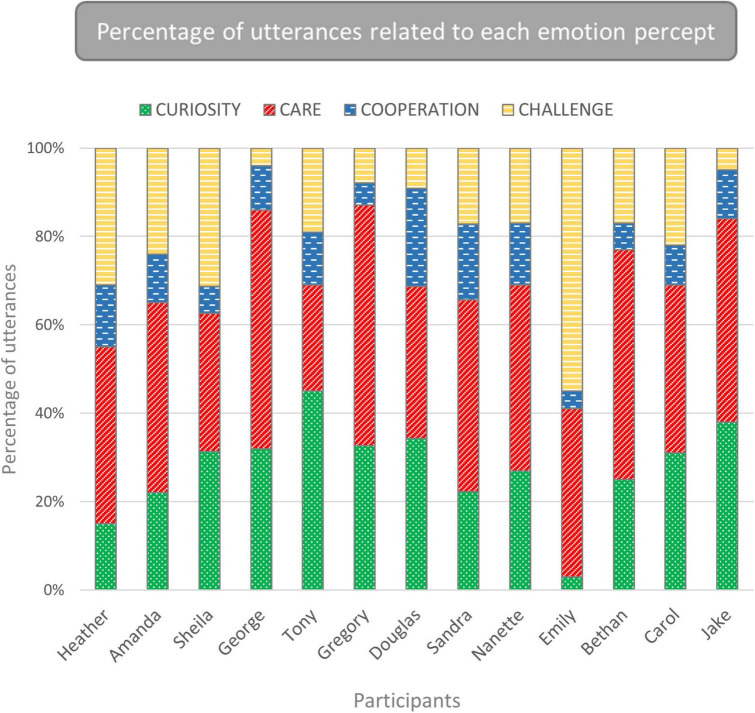
Percentages of utterances related to each of the four emotion percepts. CURIOSITY and CARE constituted between 55 and 88% of utterances for all participants except Emily.

For each participant, the number of utterances related to emotion was tallied, with subtotals calculated for each percept (Table 2), demonstrating considerable variation in the number of utterances. Nine participants made most utterances related to CARE, one made most utterances about CURIOSITY, and two made the same number of utterances related to CURIOSITY and CARE. The highest proportions of utterances were Gregory’s 55% related to CARE and Emily’s 55% related to CHALLENGE. Profiles of emotions expressed by each teacher are illustrated graphically in Figure 2, highlighting differences between individuals. Notably, for all participants other than Emily, utterances related to CURIOSITY and CARE constituted over half of the total. Table 3 gives a selection of examples of utterances for each participant.

**TABLE 2:** The total number of utterances made about emotion, and proportion of utterances related to each percept.

Total number of utterances and associated profile across emotion percepts					
Participant	Total	Curiosity	Care	Cooperation	Challenge
Heather	65	15%	**40%**	14%	31%
Amanda	46	22%	**43%**	11%	24%
Sheila	16	**31%**	**31%**	6%	**31%**
George	135	32%	**54%**	10%	4%
Tony	74	**45%**	24%	12%	19%
Gregory	40	33%	**55%**	5%	8%
Douglas	32	**34%**	**34%**	22%	9%
Sandra	23	22%	**43%**	17%	17%
Nanette	71	27%	**42%**	14%	17%
Emily	78	3%	38%	4%	**55%**
Bethan	52	25%	**52%**	6%	17%
Carol	54	31%	**38%**	9%	22%
Jake	56	38%	**46%**	11%	5%

Average	60	28%	**42%**	11%	20%

The highest proportion of utterances is highlighted in bold for each participant, and for the average.

**TABLE 3:** Examples of basic emotions for each teacher.

Examples of basic emotions		
Participant	Quote	Emotion percept(s)
Heather	(Working in a small school) we have the opportunity to have that relationship with them, to understand them and their families.	CARE
	One of the reasons I like working (here) very much is that the main focus is, if we get the relationship right then other things will follow. I’ve done things not even tenuously connected to work just to have some downtime, to have some time to build those relationships.	CARE
	(Starting the school day) that little bit later it does make all the difference, so that will be a challenge when we get back.	CHALLENGE
Amanda	The first real challenge we had was an ICT challenge because first of all we had to set up our classes… We had to set up Teams and “Show my homework” and that was a massive, massive thing, you know.	CHALLENGE
	I’ve just been with (an Advanced Higher class) on Teams and I’ve spoken to them and that’s been brilliant, but my other classes are too big to do that, so I’ve really, really found that I really, really, I really, really miss them. I miss the pupils. I miss my colleagues.	CARE, CHALLENGE
Sheila	So, we have a regularly weekly Teams meeting, keeping in touch. I’m actually really enjoying the Teams so much, from that point of view that we keep in touch by chat, the chat function on the video Team meeting. So that side of it is great.	COOPERATION
	And as to whether what I’m doing for them at the moment is impacting at all, in a good way, or not, you just don’t know…All you want is for them, to encourage them to draw kind of thing. I can offer them my time but they’re not getting the feedback and interaction.	CARE, CHALLENGE
	I’m very much of, if it’s any use to me, I’m using it. So, I embrace all technologies, but I have to admit I’m probably just a wee bit small slower in getting my head around Teams and how to use it.	CURIOSITY, CHALLENGE
George	You’ve got kids being more involved with lessons…, you’ve got enthusiasm from the kids… this stage in schooling is the part I’ve enjoyed most.	CARE
	I’ve got kids who require a good chunk of emotional support, so it’s being able to sort of provide that to them. So, it’s basically a new support system, and that for me…, was the big thing that took a lot of work, a lot of work to do.	CARE, CHALLENGE, CURIOSITY
Tony	(Microsoft training is) actually going to be really, really useful, and quite inspirational potentially because more and more of them are including educators talking about how to use it in context.	CURIOSITY
	(A community of practitioners were) at an event and talking to quite a few of them, and it was interesting because it’s perhaps a little bit of gallows humor, but we definitely felt that in many ways this was an opportunity for those of us that champion technology.	COOPERATION, CURIOSITY
	There was this positivity for me, actually, because this was an opportunity to champion something that I believed in for a long time. And it’s been very interesting, I’ve been enormously proud of how open to this my colleagues have been.	CURIOSITY, COOPERATION
Gregory	Just hearing (students’) voices is emotional…I realize how much I miss that aspect of teaching.	CARE
	It rankles me to be seen as the provider of tasks which …are providing increased anxiety	CHALLENGE
Douglas	(Being involved in a project) is what I was really very excited about getting involved in.	CURIOSITY
	I completely love my job but part of (my recovery after illness) has been the establishment of routines and ways of developing a positive outlook.	CARE (self)
	I feel I know how to (have difficult conversations). I like to help everybody when I do that. I very much enjoy when that comes together, when it is successful. I also enjoy the challenge of making it successful when it, it’s going off track, you know.	COOPERATION, CHALLENGE, CURIOSITY
Sandra	It’s hugely busy, quite stressful and I feel very responsible in my job.	CHALLENGE
	I don’t teach, but I do have a couple of groups that I do assembly with. It’s not every day but that’s nice. I like, I like that when I have that kind of contact. I’ve got my leadership team and working with them has felt amazing	CARE, COOPERATION
Nanette	I don’t think I quite realized how much time I spend interacting with the young people… The second they’re gone, it’s like, no, that’s the reason I’m a teacher. Interaction with (students) is literally what makes your day interesting and exciting and different, and you have to problem-solve left, right and centre.	CARE, CHALLENGE, CURIOSITY
	My love for outdoor education and, sort of, skills in it as well come into play and so I’ve struggled to feel like … online learning is anywhere near as effective	CURIOSITY, CHALLENGE
Emily	(Staff shortages mean) I’ve been stepping in to help. I’ve got no interest whatsoever in a promoted post, I think it’s horrific.	CHALLENGE
	I’m kind of stuck just now because I’ve got kids message me at 10 o’clock at night and I’m thinking, should I answer that or should I not? A part of me says no, but then these kids may have no one to talk to, so another part of me, like, I end up answering because I feel sorry for the kids.	CHALLENGE, CARE
Bethan	I have a lovely and committed and capable department of very young teachers who flow through this change of technology like the proverbial fish to water… if it wasn’t for help from colleague(s) I’d be really stuck.	COOPERATION CHALLENGE
	We have a wonderful, wonderful pastoral team at the school. We stay, and all the teachers stay in close contact with them…(And) my hat is off to the management team… They’ve really done an outstanding job.	COOPERATION
	I send out, you know, little personal notes to them on Google Classroom saying, hope everything is okay… Let them know in a personal touch that I’m thinking about them, but I still feel, to a degree…it eats away at me that I could be doing something else.	CARE, CHALLENGE
Carol	I took the time I had on maternity leave to think about what it was that I really wanted to do. Just over 2 years ago I moved to the post I’m in now because it’s more of what I wanted to do.	CARE (self)
	We’re used to working in small groups. We are a very, very tight team as well, so we all help each other out.	COOPERATION
	I really do miss the face to face with the pupils, I have to say. It’s, that’s why we go into the job—to be able to work with them.	CARE
Jake	(Lockdown has meant) I’ve been enabled to investigate different ways of teaching. That doesn’t mean logistically… but it’s kind of “What am I?,” not “What content am I teaching?”	CURIOSITY
	Pupils emailing me and I’m replying with, saying things like “that’s great maths but get to bed.” People say I’m crazy (for answering emails in the evenings), but I actually like it.	CARE

Emotion percepts refer to codes assigned by the authors, including where multiple emotions were identified.

Our identification of CURIOSITY was expected given previous claims within the wider literature, reinforcing the view that CURIOSITY plays an important role in teaching (Grossnickle, 2016; Ceha et al., 2019). For example, participants reported that changing circumstances had provided opportunities to pursue longstanding interests in practices, tools and technologies that they had previously never acted on, suggesting a direct link between CURIOSITY and acting with agency. Perhaps surprisingly given our *a priori* hypothesis about the important role of emotion, CURIOSITY was the dominant percept for only one participant, with considerable variability in its reported frequency (Table 2).

The most prevalent emotion reported was CARE, which was the most frequently noted percept for nine of the thirteen participants, suggesting a key role for CARE in motivating teachers’ agency. Indeed, Heather suggested this was the primary reason most teachers enter the profession. A variety of behavior was enacted in response to feelings of CARE, including presenting students with small physical tokens, some of which were practical (such as delivering jotters and pens to students’ homes), while others were symbolic (such as crocheted hearts). Other demonstrations of CARE included shifting the workday to support students working in the evenings, reassuring students of teachers’ high levels of availability, offering words of encouragement, and expressing recognition of students’ individuality and uniqueness. In addition, both Heather and Amanda made expansive embracing gestures, hugging themselves and “virtually” hugging the interviewer via their cameras, in indication of how much they missed and valued physical contact with learners.

With one exception, participants reported experiencing COOPERATION as warm feelings toward colleagues. Participants valued companionship, camaraderie and distraction provided by belonging to communities experiencing similar circumstances and with comparable goals. As well as explicit emotional support, participants recognized that sharing materials and practices also contributed to their emotional wellbeing, whether as net contributors or net beneficiaries. Another association between emotion and the experience of agency can be seen in Heather’s reporting of valuing her school’s focus on “*relationships and building those relationships and giving teachers agency to do things”* since “*if we get the relationships right then other things will follow from there.”* Here Heather directly links COOPERATION, with “doing things,” distinguishing between the emotional feeling and collective agency in action. Uniquely, the participant who reported having no professional community, either within her school or beyond, demonstrated poor emotional wellbeing. We will shortly consider the distinct circumstances reported by Emily.

All participants reported having to cope with CHALLENGE arising from their prevailing circumstances. Being unable to be physically present with students and colleagues was often experienced as distressing but was also clearly associated with agentic behavior (Sepulveda-Escobar and Morrison, 2020). Participants acknowledged emotional discomfort arising from their circumstances, then offered examples of innovative ways in which they had attempted to mitigate their difficulties through, for example, exploiting previously underutilized communication channels to make connections and share experiences with empathetic colleagues. Whilst most of our participants reported relatively few experiences of CHALLENGE despite the sudden disruption to their professional and personal lives, in one case CHALLENGE was dominant. Uniquely, 55% of Emily’s speech was focused on CHALLENGE, particularly emphasizing that she felt she was not coping, compared with others’ range of between 4 and 35%. Emily’s self-reported mental wellbeing was also notably poor, possibly as a result of the difficulties she was experiencing. She criticized herself (using “*stupid”* and “*silly”*), her students who she felt were not engaging appropriately, unsupportive colleagues, and senior management who she felt imposed unrealistic demands.

Having noted earlier how COOPERATION and agency align, here we identify another example of a link between emotion and agency. Emily reported both high levels of CHALLENGE and low levels of COOPERATION and CURIOSITY, indicating potential combinations of specific emotions, as well as links between emotions and her constrained agency. Conversely, more adaptability in agency may result from experiencing permutations of emotions that create recognizable opportunities to act in novel ways. For example, Gregory exemplified the experience of combinations of emotions when he reported CURIOSITY-driven development of expertise in creating videos that he subsequently shared online. He expressed “*a lot of satisfaction that something I spent a lot of time doing for my students* (CARE) *is actually having a wider benefit for the teaching community* (COOPERATION).”

Although presented above as distinct themes, the combination of multiple emotions and rapid switching between them was also notable, suggesting that the different percepts are typically experienced in combination or at least in rapid succession, and that emotional complexity and variation is the norm. We found support for the hypothesis that a combination of emotions influences teachers’ expression of agency and that there are individual differences in the profile of emotions teachers experience. Evidence of the fundamental nature of these differences was identifiable particularly in relation to CURIOSITY and CARE focused individuals. Quotations from two participants exemplify the distinction between the two:

*“I’ve always had an enquiring mind and a desire to, to improve what I’m doing but also just enquiring about the subjects that I’m teaching. I’m curious about mathematics, and I’m curious about how to then compare the two, mathematics and people*… *I want* (students*) to have those moments of discovery and to facilitate that*… *I always say to my pupils, you may never be passionate about maths but by the time you leave school I want you to be able to say, I know at least one person who is.”* (Gregory).

and,

*“When I started my teacher training* (the lecturer) *said ‘If you’re here because you*… *can’t wait to impart your love of Shakespeare to the young people*… *you’re here for the wrong reason. If you’re here because you like young people, because you like making connections with young people, you’re in the right place.’ And I remember thinking, thank goodness for that, I might even be able to do this after all. I think most teachers are the same. Some of them it’s very much about the love of their subject but most of us it’s just about people.”* (Heather).

Each quotation indicates an important role for emotions, respectively CURIOSITY and CARE, in teachers’ professional motivation. Whilst all participants demonstrated each percept (Table 2), indicating that all four are relevant for each of our teachers, they were reported in highly variable proportions (illustrated in Figure 2), suggesting distinct individual differences in the ways that participants experience and act on emotions associated with their professional life (see Table 3 for more exemplars).

## Discussion

Shortly after the covid pandemic was declared, we interviewed thirteen teachers working in Scottish secondary schools. Thematic analysis of interview transcriptions confirmed assumptions related to professional agency that teachers would take volitional action in response to prevailing exigencies. We identified four sub-themes of agency: adaptability, collective agency, constrained agency, and inaction. We found evidence in support of the assumption that teachers would report feelings of CURIOSITY in respect of their professional lives, and we identified three other emotion percepts participants reported as important to their commitment as teachers, these were CARE, COOPERATION and CHALLENGE. Further, we found that the four emotions were experienced in different proportions between individuals, and that they were frequently reported together.

Contemporary approaches to teaching reform such as Scotland’s Curriculum for Excellence emphasize the role of teachers in developing and delivering content in the context of their unique classroom situations in a climate of reflexivity and cooperation between classroom practitioners exercising their professional agency (cf. Scottish Executive, 2006a). In emphasizing flexibility and adaptability, contemporary teaching approaches aim to exploit the complexity arising from individual differences in teachers’ attributes, knowledge and experience, rather than imposing uniformity on teaching practice. To date, however, exploration of individual differences in teachers’ basic emotions and their experience and expression of agency has been limited. The current study demonstrates teachers acting with agency in response to the unprecedented circumstances unfolding at the time the interviews were conducted, and highlights the relevance of basic emotions, albeit in differing proportions, in achieving that agency.

The project to explore emotions relevant to teachers’ experience of agency was conceived before the start of the pandemic, and it is evident that the challenges arising from the unprecedented circumstances impacted the results we found. Practically, teachers had to deal with pupils who lacked digital connections required for remote learning. Socially, they were supporting those living in poverty in inadequate housing. Emotionally, they were addressing mental health difficulties in themselves and their learners (Moss et al., 2021). The prevalence of CARE toward learners in the circumstances prevailing at the time of data collection is perhaps unsurprising given teachers’ pastoral role in supporting student wellbeing and the considerable threat to mental health resulting from the experience of a pandemic. Although data about teachers’ responses to the pandemic are still scarce, a recent survey found an association between teachers’ wellbeing and the support they felt from friends, colleagues and senior management (Connor et al., 2022). This association aligns with our findings of the importance of teachers feeling CARED *for* and recognizing that they have opportunities for COOPERATION with others. It also aligns with the experience of collective agency, in which colleagues provide practical support in tackling CHALLENGING situations.

### Implications for theory and practice

Distinct from existing research that offers cognitive rationalizations for teacher agency such as serving society, on the basis of the current findings we make a novel proposal for four emotion percepts that influence the experience and expression of agency. Our proposal is also distinct from research exploring secondary emotions arising from appraisals of teachers own performance or that of their students by its focus on basic emotions preceding such appraisals.

We have reported connections between emotions and teacher agency, both of which are recognized as complex constructs. Complex systems operate on multiple levels, across different temporal scales, and are liable to be impacted in non-linear ways by myriad factors (Overton, 2014; Wilson, 2016). A corollary of recognizing an area of study as complex is acknowledging that investigating that domain solely by identifying and measuring variables will generate little insight (Lykken, 1991; Agar, 2004; Gigerenzer, 2010). Our methodological choices recognize that phenomenological and methodological issues constrain how research is conducted within and between complex and dynamic systems, and further, that there is no single effective approach to studying complex systems. Instead, research methods that recognize subtle and complex interactions that influence teachers’ agency are preferable to attempting to establish categorical distinctions such as assuming that teachers are *either* CURIOUS *or* CARING *or* COOPERATIVE *or* CHALLENGED. Our open process of analysis proved particularly appropriate for identifying complex patterns of interaction between emotions and feelings of agency.

Paradoxically, complex systems can be described by a few simple rules governing how component entities interact to generate a wide range of behaviors at higher levels of the system (Wilson, 2016). The challenge is to accurately identify and describe elemental components, together with relevant rules under which they operate. In response to calls for psychology to mature as a discipline through greater theory development (Borghi and Fini, 2019), we therefore propose four theoretical rules that aim to articulate teachers’ experience of agency, in particular extending existing theories that omit or downplay references to emotions (Emirbayer and Mische, 1998; Priestley et al., 2015; Aspbury-Miyanishi, 2022). The first three encapsulate results and discussions already reported above: Proposition (1) *four emotion percepts of CARE, CURIOSITY, COOPERATION, and CHALLENGE substantially influence teachers’ voluntary motivated behavior;* Proposition (2) *individual differences between teachers are reflected in the amount and proportion of emotions reported;* Proposition (3) *the four emotion percepts are experienced by engaged teachers concurrently or in rapid succession.*

In recognition of the unique circumstances of heightened concern about students’ wellbeing, we offer Proposition (4) *prior experiences and the exigencies of the current situation influence which category of action has priority in the moment*. For example, although in the current situation he expressed a greater emphasis on CARE, Gregory reported an earlier episode when he was driven by CURIOSITY to gain skills in digital learning. While proposition 3 highlights concurrency of emotions, we also recognize that proportions of each percept may change over time for an individual. For instance, a similar data-gathering exercise at another time may have seen the same participants reporting more CURIOSITY and COOPERATION, and less emphasis on CARE.

As well as novel theoretical propositions, we highlight three distinct features of our approach. The first is the contrast with existing theories of teacher emotions. We examined the role that emotions play in *producing* behaviors, in contrast with the prevailing focus on emotions that *result from* appraisal of actions (Pekrun, 2006; Pekrun et al., 2007; Frenzel, 2014; Pekrun and Perry, 2014). Other research has identified “strong” emotions as relevant to educational practices (Danvers, 2015; Tillmanns, 2019), and CURIOSITY specifically has been shown to relate to outcomes (Silvia, 2008; Grossnickle, 2016). Here we have offered greater specificity about “strong” emotions, presenting evidence that teacher agency can be understood as a behavioral outcome motivated by CURIOSITY, as well as three additional emotion percepts of CARE, COOPERATION, and CHALLENGE.

The second distinct feature of our approach is the contrast with the existing body of research focused on teachers’ negative emotions, such as UK teachers reporting high levels of stress, with symptoms of poor wellbeing attributed to high workload and lack of support (Education Support, 2020). Stress leads to decreased motivation, absenteeism, poor student behavior, and lower academic achievement (Ravalier and Walsh, 2017). Particularly in Emily’s case we found evidence supportive of an association between negative emotions, wellbeing, and the experience of agency. However, an inference from the focus on negative emotions (Frenzel, 2014; Pekrun and Perry, 2014) might be that these exert *more* influence on teachers’ motivation than positive emotions. In contrast with Emily though, other participants did not report feeling overwhelmed by the CHALLENGE they were experiencing, but rather appeared able to balance the influence of positive and negatively valenced emotions in their experience of agency. Further effort to gain understanding of interactions between emotions of different valence is needed, particularly given findings that positive engagement is more conducive to favorable outcomes than punishment (Skinner, 1953; Prescott and Buchanan-Smith, 2003).

### Limitations and opportunities

As we highlighted in the introduction, there is ongoing ideological debate about the nature of emotions, specifically whether there are “basic” emotions, their nature and number, and how they might relate to secondary emotions (Barrett et al., 2007; Chen, 2016; Panksepp et al., 2017). Researchers distinguish between innate, phylogenetically developed basic emotions (Damasio, 2006) and secondary emotions characterized by the degree of cognitive processing associated with their experience (Leyens et al., 2001; Becker-Asano and Wachsmuth, 2008). For instance, the experience of enjoyment is reported as “*resulting from”* historical actions, current engagement or future anticipation (Frenzel, 2014, p. 495, italics added). Given that a consensus is unlikely to emerge anytime soon between opposing viewpoints, we limit our current position to a pragmatic proposal that the four percepts at least satisfy the categorical and psychological definitions outlined by Celeghin et al. (2017), and therefore offer potentially useful distinctions (Box, 1979). Future interdisciplinary efforts to clarify definitions, distinctions, phylogenesis and necessary and sufficient components of emotions would prove valuable. Similarly, the current paper proposes no physiological mechanisms or emotional/cognitive processes linking the four percepts with agency. A starting point could be work characterizing CURIOSITY as the inherent reward experienced when acquiring knowledge, leading to a cycle of information-seeking behavior and increasing knowledge (Murayama, 2022). We are unaware of similar work linking multiple basic emotions. In order to record relevant behavioral and physiological measures of emotion in the classroom, recent developments in cognitive neuroscience (cf. the mobile cognition approach: Ladouce et al., 2017) indicate how future studies may augment understanding by include measures such as heart rate variability or mobile EEG recordings.

We present a qualitative study, which remains relatively unusual in a discipline that favors self-report measures (Dinsmore et al., 2008; Dinsmore, 2017). Of course, the nature of complex systems necessarily requires dynamism and flexibility throughout the analysis process, as well as recognition that a variety of means will be needed to guard against spurious associations, lack of reproducibility and future replication failures (Wasserstein and Lazar, 2016). As psychology transitions from assumptions that behavior is purely rational, to acknowledging that motivated behavior incorporates emotion (Hall and Knapp, 2015) opportunities arise to incorporate multiple disciplinary and conceptual foci in future efforts to understand complex psychological processes. We advocate proceeding cautiously using appropriate methods to extend understanding of the impact of emotion percepts in catalyzing effective teaching. In particular, relationships between researchers and the objects of investigation need to be explicit and subject to scrutiny, with a range of methods used to improve validity and triangulation of findings.

Although we have responded to ongoing encouragement to vary methodological approaches (e.g., Lykken, 1991; Borsboom et al., 2009; Gigerenzer, 2010), we recognize that single engagements with participants do not present the same opportunities to record change over time that longitudinal and ethnographic methods would have afforded (Agar, 2004; Dinsmore et al., 2008). While participants in the present study reported major recent changes, conducting subsequent studies over extended timeframes might enhance the validity of reports by allowing intraindividual comparisons of multiple observations.

Also related to methodological concerns, we identified instances of gesture conveying supplementary information about participants’ emotional states (Hostetter, 2011). To our surprise, reporting of such additional data is often missing from relevant literature. For example, Oplatka (2012) reports only the words of teacher interviews. Similarly, although Eidsvåg and Rosell (2021) report actions, their analysis to “make sense of what we perceived was happening” (p. 88) results in summary explanations such as reporting that a participant’s “body language is vigorous and confident” (p. 91), rather than describing specific gestures and movements. Aligned with concerns about the universality of facial expressions conveying specific emotions (Feldman-Barrett, 2017), there are concerns about the ubiquity of specific gestures communicating additional information (Hostetter, 2011) and a pressing need to develop effective coding strategies to facilitate robust interpretation of gestures. Nonetheless, we encourage the reporting of non-verbal communication and physiological measures alongside verbal responses as a way of generating richer data leading to more complete understanding.

It is also important to acknowledge that the current study was unable to determine the extent to which lack of CURIOSITY, CARE, COOPERATION, and excessive CHALLENGE each influenced constrained agency. Indeed, further studies are needed to establish whether the four percepts provide a complete account of the emotional experience associated with agency. For example, it may be that teacher agency is enhanced or compromised by the experience of specific profiles of emotional percepts. We also note that although there is widespread adoption of the idea that agency is an important feature of effective teaching practice, to date there is relatively limited empirical evidence regarding how agency operates in the classroom, whether it can be developed, and how it changes with experience. Further enquiry is clearly necessary and we particularly advocate proceeding cautiously before encouraging widespread adoption of pedagogical practices that lack strong evidential support (Newton and Salvi, 2020).

The current study involved only teachers working in the secondary stage, giving rise to obvious opportunities to examine further questions. Do teachers in the primary stage show similar sub-themes of agency? Do tertiary-stage teachers have different experiences of basic emotions? Do teachers of different subjects have distinct differences in their basic emotions? Are there different basic emotions at work for some teachers compared to those already identified in the current paper? We also recognize the need to investigate the roles of students and the wider social context in teachers’ experience of agency and the efficacy of their practices (Priestley et al., 2015). Moreover, participants in the current study volunteered their efforts at a time when teachers were experiencing unprecedented disruption. They may therefore have been unrepresentative of the profession in judging that they had capacity for additional effort, whilst others might have felt that their ongoing volume of work and personal circumstances precluded their participation. Future research should also examine effects of students’ and teachers’ emotions on student satisfaction, wellbeing and academic achievement (Collie et al., 2017). More broadly, work in classrooms could inform understanding about whether each of the four percepts is always necessary for teachers to experience agency, why some teachers feel overwhelmed by professional and personal circumstances affecting their work, and whether students and teachers necessarily experience interactions in the same way (for example, do teachers’ expressions of CARE sometimes translate into experiences of CHALLENGE for students?) Likewise, since organizational and environmental factors affect individuals’ development and enactment of agency (Hökkä et al., 2019), pressure to achieve centrally imposed targets and increase efficiency might have significant impact on teachers’ capacity to experiment with innovative practices and pedagogical resources. Since context-dependent curricula recognize teachers’ individual differences and focus on their critical thinking skills and reflectivity to promote increased self-awareness (Education Scotland, 2021), increased awareness of the role of emotions in teaching may support relevant institutions in developing appropriate curricula and monitoring learning outcomes.

Coaching focused on emotional awareness has been shown to impact the sense of personal agency experienced by managerial staff (Hökkä et al., 2019). Adopting a similar approach using longitudinal and comparative studies would provide invaluable insights into the impact of teachers’ awareness of emotion percepts on motivation, self-efficacy, engagement, behavior, and teaching outcomes (Reeve and Shin, 2020; Usher, 2021). We are optimistic about opportunities and outcomes arising from greater focus on emotions, particularly in teachers’ effective modeling of emotion percepts and associated behaviors for students in the same way that they model meta-cognitive abilities (cf. Branigan and Donaldson, 2019, 2020). Partnerships between psychology and education practitioner-researchers should also encourage more nuanced development and adoption of research findings in education settings (Immordino-Yang, 2016), particularly exploring the impact and limitations of awareness of emotion percepts on pedagogical practices through collection and analysis of rich, robust data (Newton and Salvi, 2020).

## Conclusion

We examined teachers’ professional agency through enthusiastic and effortful commitment to adapting their behaviors as circumstances demanded, to support themselves, their colleagues and their students. We clarified existing conceptualizations of agency by identifying that teachers act adaptably at multiple levels, making strategic, tactical and operational choices, including choices not to act. We also identified constrained agency when choices felt limited, leading to action without wholehearted engagement, as well as agency operating within communities of teachers acting collectively. In questioning “*what motivates us to teach,”* we identified factors linked to basic emotions and proposed a novel theory that teachers’ agency is substantially influenced by feelings of CARE, CURIOSITY, COOPERATION, and CHALLENGE. We also proposed that the degree to which these emotions are experienced differs between individuals, that the percepts are experienced concurrently or in rapid succession, and that circumstances influence which emotions are most influential in any situation. We distinguish basic emotions that motivate engagement in teaching from achievement emotions that result from such commitment. For example, we suggest that feelings of CARE that motivate a teacher to spending additional time with students is more basic than the pride that results from students’ success at reaching goals, although the potential link between the two is evident.

At a time of major challenges, participants in the current study showed how dedication to their craft, students, and colleagues led them to volitional actions aimed at minimizing losses and exploiting opportunities for the perceived benefit of each of those populations. Those concerned with whether students reliably experience supportive teaching may be reassured by our findings of highly motivated, agentic teachers. Further careful and critical consideration of the role of emotion percepts may also help educators to fine-tune responses to their own and their students’ individual needs. Indeed, our view is that gaining a clearer understanding of the role that emotions play in the classroom will encourage learner-shaped education that promotes the development of agency, instead of attempting to mold compliant school-shaped learners. By listening to and observing teachers we can see that implicit awareness of emotion percepts already supports agency and **good** pedagogy. Our hope is that future studies will show that enhancing teachers’ explicit understanding of the roles of CARE, CURIOSITY, COOPERATION, and CHALLENGE in their experience of agency will result in more **great** practice.

## Data availability statement

The raw data supporting the conclusions of this article will be made available by the authors, without undue reservation.

## Ethics statement

The studies involving human participants were reviewed and approved by the School of Psychology and Neuroscience Ethics Committee, University of St. Andrews. The participants either provided written informed consent to participate in this study or gave verbal informed consent at the start of the interview.

## Author contributions

KP: conceptualization, methodology, project administration, formal analysis, investigation, resources, data curation, writing—original draft, visualization, writing—review and editing, and funding acquisition. PM: conceptualization, methodology, writing—review and editing, and supervision. DD: conceptualization, methodology, project administration, visualization, writing—review and editing, supervision, and funding acquisition. All authors contributed to the article and approved the submitted version.
