# Supplementary material for: Teachers’ emotions in the time of COVID: Thematic analysis of interview data reveals drivers of professional agency
Source: Front Psychol. 2022 Nov 3;13:987690. doi: 10.3389/fpsyg.2022.987690 (PMC9670191; doi:10.3389/fpsyg.2022.987690)
Supplement: Supplementary file 1 [file Data_Sheet_1.docx]

# Appendix A. Topic Guide

The following document was used as a topic guide to inform the range of discussions in interviews that took part between Karen Porter and participants in the study described in the preceding journal article.

## Introduction

My name is Karen Porter, and I am a PhD student at the University of St Andrews, funded by the Economic and Social Research Council through the Scottish Graduate School of Social Sciences. My studies are focused on teachers. I am fascinated by how people and groups handle challenging situations, and the current COVID19 is going to be the most challenging time that many of us will experience in our lives. I’m particularly interested in how teachers will rise to the challenges, and how their responses might change teaching practice in the future.

In this interview I would like to gain an understanding of how the current health crisis is affecting your actions, thoughts and emotions in your role as a teacher. There is no pressure or need for you to talk about any specific topic, and if at any time you wish to stop or change subjects, I will be happy to be led by you in order that you are comfortable with our discussion.

With your permission, I would like to make notes as we go along, and I would also like to record our session.

## Indicative questions

| Q1 | Are you happy for me to:   - record our interview - make notes | Yes / No  Yes / No |
| --- | --- | --- |

To begin with, I would like to find out a bit about you.

| Q2 | Can you tell me a bit about you:   - your name and age (if you’re comfortable giving that) - how long you have been teaching - what and where you teach - what posts you hold (eg probationer teacher, classroom teacher, head/depute head of department, head/depute head of year, member of SMT) - if you are currently actively seeking promotion or a professional move, what steps are you taking towards that goal |
| --- | --- |

Next I would like to explore your responses to the changes that have suddenly arisen as a result of the coronavirus epidemic that has been developing and impacting your personal and professional life

| Q3 | How have you responded to the recent crisis, in your thoughts, behaviour and feelings?  **Thoughts**   - What consideration have you given to what is **possible** and what is no longer possible in the changing circumstances? - Have you considered what is **desirable** and no longer desirable from you as a teacher, and how this might inform what you do and how? - How have you considered risks arising from the new circumstances? - Have you identified new opportunities? - Have you considered the personal resources available to you to perform effectively in the current environment?   - Resources might include skills, training and previous experiences.   - Aspects of your personality   - Your upbringing and current family situation - How labels you might have applied to yourself as a teacher are relevant now, or have changed. Examples: competent, novice, dynamic, steady, enquiring, masterful. - Have your values and beliefs changed or been brought into sharper focus, and if so, has awareness of your values and beliefs altered your teaching? |
| --- | --- |

| Q3 | **Actions / behaviour**   - In what ways have your actions changed recently, and how have they remained the same or similar to previously? - How have you decided what to change and what to keep doing the same?   - Have you broken with established norms of behaviour? In what ways? - How have you chosen the resources and materials you have been using recently? Resources might include teaching materials, technology for delivering lessons, your workspace, infrastructure and location   - How have choices been informed by your judgements about what is most likely to be effective? - Have you developed new skills and knowledge in order to continue to teach effectively? What prompted you to do so? - Have you used skills you learned in other contexts in your teaching recently that you wouldn’t previously have used? - What have you been doing to ensure you can be an effective teacher? Have you chosen those activities deliberately for the purpose of self-care? - Has your use of language changed, for example your use of personal or emotional language? |
| --- | --- |

| Q3 | **Affect**   - What have been your general moods in recent weeks? - How have your emotions been affected by the professional challenges you have experienced? - What is your recent experience of levels of energy and ability to focus? - How has your self-evaluation been affected? Evaluations might include competence, autonomy, resilience and capacity to face challenging circumstances. - What has been your experience of **providing** social support?   - colleagues and school management team   - Family and friends   - Students   - other significant social factors - What has been your experience of **receiving** social support?   - colleagues and school management team   - Family and friends   - Students   - other significant social factors - What **changes** have you noticed in your mood and emotions over time, comparing pre-C19 with early-C19 and ongoing C19? |
| --- | --- |

Future engagement with the current research project

| Q4 | Would you be willing to be contacted by Karen Porter in the future about further opportunities to take part in research into how teachers are coping with the need to make changes in their teaching practice?  A possible follow up would be to invite teachers to work in a small team of co-researchers investigating psychological factors that support effective teaching and learning. Might you find participating in such a research project of interest? | Yes / No  Yes / No |
| --- | --- | --- |
